# Fatigue Testing of Wearable Sensing Technologies: Issues and Opportunities

**DOI:** 10.3390/ma14154070

**Published:** 2021-07-21

**Authors:** Andrea Karen Persons, John E. Ball, Charles Freeman, David M. Macias, Chartrisa LaShan Simpson, Brian K. Smith, Reuben F. Burch V.

**Affiliations:** 1 Department of Agricultural and Biological Engineering, Mississippi State University, 130 Creelman Street, Starkville, MS 39762, USA; akp4@msstate.edu (A.K.P.); clsimpson@abe.msstate.edu (C.L.S.); 2 Human Factors and Athlete Engineering, Center for Advanced Vehicular Systems, Mississippi State University, 200 Research Boulevard, Starkville, MS 39759, USA; jeball@ece.msstate.edu; 3 Department of Electrical and Computer Engineering, Mississippi State University, 406 Hardy Road, Starkville, MS 39762, USA; 4 School of Human Sciences, Mississippi State University, 255 Tracy Drive, Starkville, MS 39762, USA; cfreeman@humansci.msstate.edu; 5 Department of Kinesiology, Mississippi State University, P.O. Box 6186, Starkville, MS 39762, USA; dmacias@columbusortho.com; 6 Columbus Orthopaedic Clinic, 670 Leigh Drive, Columbus, MS 39705, USA; 7 Department of Industrial and Systems Engineering, Mississippi State University, 479-2 Hardy Road, Starkville, MS 39762, USA; smith@ise.msstate.edu

**Keywords:** fatigue testing, cyclic testing, low-cycle fatigue, high-cycle fatigue, wearables, lead failure, stretch sensor, hysteresis, cyclic softening, fatigue testing standards

## Abstract

Standards for the fatigue testing of wearable sensing technologies are lacking. The majority of published fatigue tests for wearable sensors are performed on proof-of-concept stretch sensors fabricated from a variety of materials. Due to their flexibility and stretchability, polymers are often used in the fabrication of wearable sensors. Other materials, including textiles, carbon nanotubes, graphene, and conductive metals or inks, may be used in conjunction with polymers to fabricate wearable sensors. Depending on the combination of the materials used, the fatigue behaviors of wearable sensors can vary. Additionally, fatigue testing methodologies for the sensors also vary, with most tests focusing only on the low-cycle fatigue (LCF) regime, and few sensors are cycled until failure or runout are achieved. Fatigue life predictions of wearable sensors are also lacking. These issues make direct comparisons of wearable sensors difficult. To facilitate direct comparisons of wearable sensors and to move proof-of-concept sensors from “bench to bedside”, fatigue testing standards should be established. Further, both high-cycle fatigue (HCF) and failure data are needed to determine the appropriateness in the use, modification, development, and validation of fatigue life prediction models and to further the understanding of how cracks initiate and propagate in wearable sensing technologies.

## 1. Introduction

Interest in wearable stretch sensors has increased due to their potential uses in medical applications to monitor the health of a patient [1,2,3,4,5,6,7,8,9,10], to assess biomechanics, [11,12,13,14,15,16,17,18,19,20,21], and as drug delivery systems in pharmaceutical applications [22,23]. Wearable sensors may also have applications in athletics. [11,18,21,24,25,26,27,28], soft robotics [21,29,30], ergonomic assessments [19] and deep space exploration [31]. This interest is especially timely as the SARS-CoV-2 (COVID-19) epidemic has led to decreased in-person office visits to medical professionals while concomitantly increasing the number of virtual visits via telemedical platforms [32,33,34]. The increased use of telemedicine has led to an increased interest in the use of wearable sensors to monitor the health of patients outside of the clinical setting [18,35,36]. By 2022, over 1,000,000,000 wearables are expected to be in use globally [37], and although the opportunities provided by wearable sensors are recognized, few sensors have been formally validated [38], and research is still needed to determine the accuracy, interpretation, and applicability of the data provided by wearable sensors [38,39]. While many wearable sensor prototypes have been described for the previously mentioned applications, for these sensors to move from “bench to bedside”, standardized testing methods, including those that focus on the fatigue life of a wearable sensor, are needed [40].

**Figure 1:**
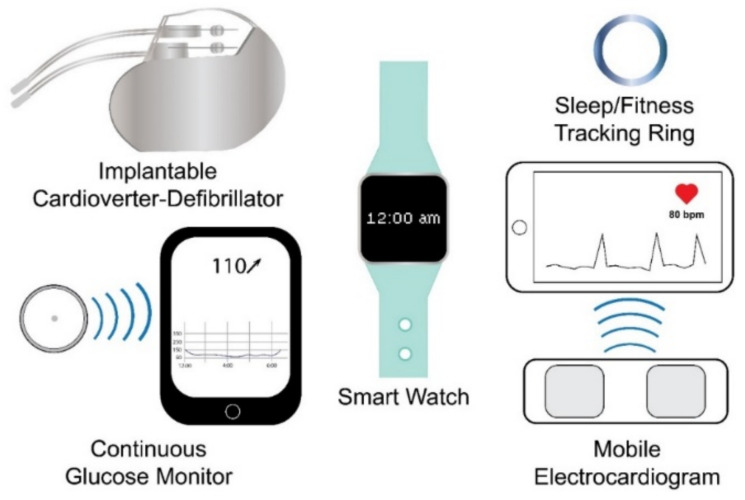
Examples of wearable sensing technologies. Wearable sensing technologies include internal (e.g., internal cardioverter-defibrillator and continuous glucose monitor) and external (e.g., smart watch, sleep/fitness tracking ring, and mobile electrocardiogram) wearable sensing technologies.

Wearable sensing technology may be broadly defined as electronic devices embedded within or worn upon the body that rely on sensors to capture and transmit data to an integrated display unit, a computer, or a smartphone [39,41,42,43,44]. Based on this definition, currently marketed wearable sensor technologies can be divided into internal sensors that are subcutaneously implanted in the body and external sensors that are worn on the body. Examples of the former include implantable cardioverter defibrillators (ICD), which sense cardiac depolarization [45,46,47,48,49,50,51,52,53,54,55,56,57]; implantable loop recorders, which are implantable electrocardiograms (EKG or ECG) [22,23]; and invasive continuous glucose monitors (CGM), which use a sensor embedded in the upper arm, abdomen, or gluteus to measure glucose levels from interstitial fluid [58,59], while examples of wearables that fall into the latter include smart watches, sleep and fitness trackers, or ECG sensors (Figure 1). Fatigue failures of wearables, such as ICDs, are widely recognized and result in significant morbidity and mortality [45,46,47,48,49,50,51,52,53,54,55,56,57]; therefore, understanding the electromechanical fatigue and failure properties of proposed wearable sensors is paramount in insuring patient safety. Although such failures are recognized with internally implanted sensors, a lack of standardized fatigue testing methods and validation studies [38,40], coupled with sensors fabricated from varying materials, has led to a paucity of comparable data regarding the durability and accuracy of wearable sensors. Further, the fatigue testing of wearable sensors is confounded due to the need to predict not only the fatigue life of the materials comprising the sensor but also the fatigue life (stability) of the signal produced by the sensor.

## 2. Internal Wearable Sensing Technologies

Internal wearable sensing technologies are fabricated from inert materials that do not elicit a bioreactive response upon implantation. Implantable materials include metals such as stainless steel and titanium, polymers such as polyethylene (PE) and polymethylmethacrylate (PMMA), and ceramics such as hydroxyapatite [60]. Within the United States, implanted medical devices, including ICDs, internal loop recorders, and CGMs, which may also be considered wearable technologies, are regulated by the Food and Drug Administration (FDA) as Class III medical devices [61,62,63]. For internal wearable sensing technologies to receive FDA approval to enter the marketplace, the sensor must either receive Premarket Approval (PMA) by undergoing clinical trials to demonstrate its safety and efficacy, or the sensor may be cleared to enter the market by the Premarket Notification 510(k) process which involves submitting premarket data to the FDA to show that the proposed internal sensor is “substantially equivalent” in both safety and efficiency to a sensor that did not require PMA approval and is already on the market [61,62,63,64,65]; however, “substantially equivalent” devices may be fabricated using both differing materials and differing mechanisms of action providing the safety profiles of the two devices are similar [61], but because of the variation in materials and mechanisms of action, testing standards that apply to one device do not necessarily correlate with the comparative device [61]. Further, incremental changes to medical devices initially approved by the PMA process may be submitted to the FDA for market clearance via a supplement that may not require clinical data [48,49], and underlying issues with the updated device may not be recognized until the device is in widespread use [61]. In contrast, the European Union has recently implemented regulations that require clinical evaluations for implantable medical devices throughout the lifespan of the device; therefore, clinical data is required from pre-market evaluations to post-market evaluations. Further, clinical data is required even in the case of incremental changes [66,67]. For example, in the United States, updated ICD leads can enter the marketplace through the use of a PMA supplement that does not include clinical data demonstrating the safety and efficacy of the updated lead; whereas, under the new regulations adopted by the European Union, the introduction of updated pacemaker leads requires clinical data that address the safety and efficacy of the lead [48,49,66,67]. Fatigue failures of ICD leads approved via PMA supplementation in the United States are common [45,46,47,48,49,50,51,52,53,54,55,56,57], but whether the new European Union regulations either prevent the entry of such leads into the market or prevent adverse outcomes by the timely recognition of issues with the leads during post-market surveillance remains unclear [68].

**Figure 2:**
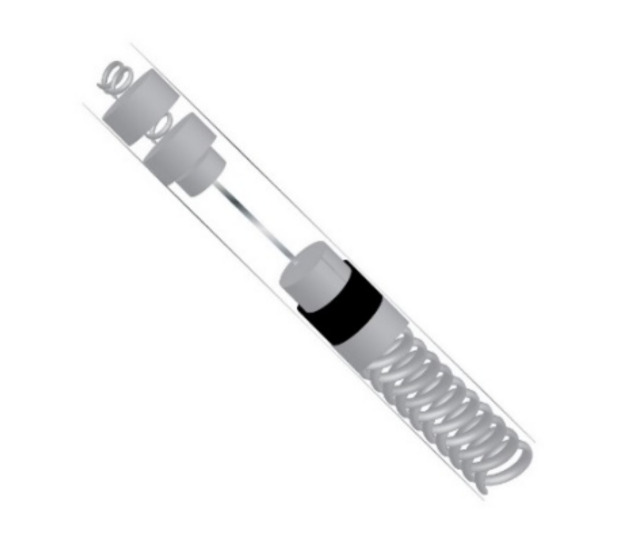
Simplified cross-section of an implantable cardioverter-defibrillator lead.

ICD leads are typically comprised of either a low-voltage, nickel–cobalt–chromium–molybdenum alloy coil conductor or a high-voltage, silver or platinum coil conductor that is coated with ethylene tetrafluoroethylene and poly-tetrafluoroethylene and housed within a silicone cylinder that also acts as insulation to separate the conductive cables from the electrode tips [53,69,70] (Figure 2). Leads are thin and flexible, ranging in diameter from 2.1 to 2.87 mm, to navigate the vasculature and are inserted into the myocardium [69,71]. Electrodes at the tips of ICD leads act as sensors to recognize atrial and ventricular depolarizations [71]. Upon sensing a depolarization event, a signal is sent to the pulse generator, which contains a battery and a circuit board, where the signal is processed, allowing for both the detection and correction of abnormal heart rates and rhythms based on programmable thresholds [71]; therefore, ICD leads are critical sensing mechanisms, and preventing fatigue failures of the leads will prevent adverse patient outcomes.

ICD leads are subjected to more than 100,000 cycles of flexure per day [53], and fatigue failures of ICD leads may result in high impedance if the lead is fractured or low impedance if the insulation fails, causing the lead to short circuit [70]. Additionally, fatigue failures of ICD leads may result in noise in the signal [54,55,57,70], inappropriate pacing [45,69], inappropriate defibrillation [45], the delivery of unnecessary shocks resulting from oversensing [45,46,50], or mortality [46,47], especially in a failure to detect ventricular fibrillation [71].

Few published studies have assessed the fatigue life of ICD leads [72]; however, Altman et al. [72] have noted that how the interaction between the coil and the individual wires comprising the coil affect the fatigue life of the lead is unknown and may require special consideration when evaluating fatiguing methods. Liu et al. [73] found that the stresses placed on the lead in vivo could be determined by applying classic mechanical principles to a model created from 3D images rendered from angiograms and argue that this method can facilitate fatigue-life predictions of the leads. Recently, due to the ongoing high incidence of failure, standard protocols for the fatigue testing of ICD leads have been proposed [74]. The proposed method involves the application of a buckling or a bending force at a rate of 5 Hz to 12 samples per four curvature amplitudes of 0.78 cm^−1^, 1.11 cm^−1^, 2.12 cm^−1^, and 2.45 cm^−1^ (*n* = 48). All tests are performed at a temperature of 23 ± 5 °C. The first 1000 cycles are considered run-in, and testing is terminated upon failure of the lead or after the completion of 5,000,000 cycles. Disruption of electrical continuity or a 150% rise in the resistance over the initial resistance value constitutes failure [74]. While researchers are working to standardize testing methodologies for some internal wearable sensors [74], fatigue testing methods for external wearable sensors are not standardized [40], and a variety of testing methodologies, rates, and cycling regimes have been reported in the fatigue testing of proposed wearable sensors [6,11,12,13,20,21,30,75,76,77,78,79,80,81,82,83,84,85,86,87,88,89,90,91,92,93,94,95,96,97,98,99,100].

## 3. External Wearable Sensing Technologies

Many external wearable sensor “proofs-of-concept” have been proposed to monitor health [1,2,3,4,5,6,7,8,11,12,13,14,15,16,17,18,19,20,21,22,23], to act as drug delivery systems [2,22,23], or to improve athletic performance [11,18,21,24,25,26,27,28]. Some of the proposed sensors have been subjected to fatigue testing; however, the methods and materials used for testing have varied. Of those sensors subjected to fatigue testing, the majority have been wearable stretch sensors. For stretch sensors to be successfully implemented in such applications, the sensors should have four basic properties which include: (1) being flexible/stretchable, (2) exhibiting both a rapid response and recovery time (low electrical and mechanical hysteresis), (3) producing a linear relationship between strain and resistance with low electrical drift to prevent noise in the signal that may result from upwards or downwards changes between the base and the peak resistances across the intended life-cycle or use for the sensor [11,21,100,101,102], and (4) the sensors must be fatigue-resistant. 

To meet these desired properties, polymers including polytetrafluoroethylene (PTFE) [13], polyurethane acrylate (PUA) [80], polydimethylsiloxane (PDMS) [81,85,89,100], polyimide (PI) [82,83], poly(3,4-ethylenedioxythiophene) (PEDOT) [82,96,99], poly(styrenesulfonate) [82], silicone [88,90], thermoplastic urethane [99,103], ionogels [97], and hydrogels [21,91], knitted and woven textiles [76,90,95,98,104], overlock stitched textiles [78], conductive multifilaments that can be incorporated into textiles [92], polymer coated textiles [96], sensors embedded in apparel [12], carbon nanotubes (CNTs) [20,75,77,79,87,93,105], graphene [30,84,94,106], or combinations thereof are often used in conjunction with conductive materials. 

Most studies that assess the fatigue of wearable stress sensors employ high-amplitude, low-cycle fatigue (LCF) of ≤ 10,000 cycles [107,108] that typically does not result in failure of the sensors [1,11,12,13,21,76,78,79,80,81,84,85,87,88,89,90,92,93,94,95,96,97,98,103,104]; therefore, the number of cycles to failure (*N_f_*) are unknown. To reiterate, the purpose of such studies is to demonstrate “proof of concept” for a given sensor, not to necessarily identify the mechanical properties of the materials used in the fabrication of the sensor; however, for physiologically based applications, such as cardiovascular monitoring or measuring the typical range of motion for a joint, the sensors need to withstand low-amplitude, high-cycle fatigue (HCF) [109]. For a stretch-sensor-based sports application example, in the United States, collegiate basketball players take an estimated 1260 running steps in a game [110]. Based on the 2019–2020 regular-season schedule of a National Collegiate Athletics Association (NCAA) Division I (D1) basketball team, a sensor would undergo approximately 40,320 cycles across 32 games, which would increase with post-season play in the Southeastern Conference (SEC) or NCAA tournaments. In contrast, soccer midfielders take approximately 8910 steps during a match [111], which, based on a 2019 NCAA D1 women’s soccer schedule of 20 games, would result in approximately 178,200 steps in a season. 

**Figure 3:**
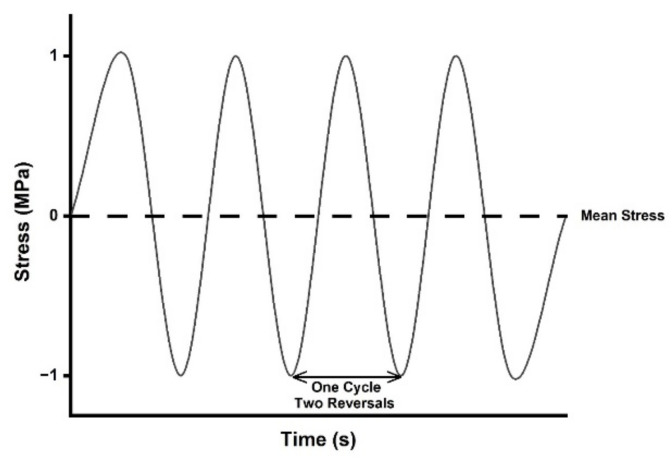
Sinusoidal pattern created by fully reversed loading. The sinusoidal pattern results from the alternation of tensile (reversal 1) and compressive (reversal 2) loading during cycling.

**Figure 4:**
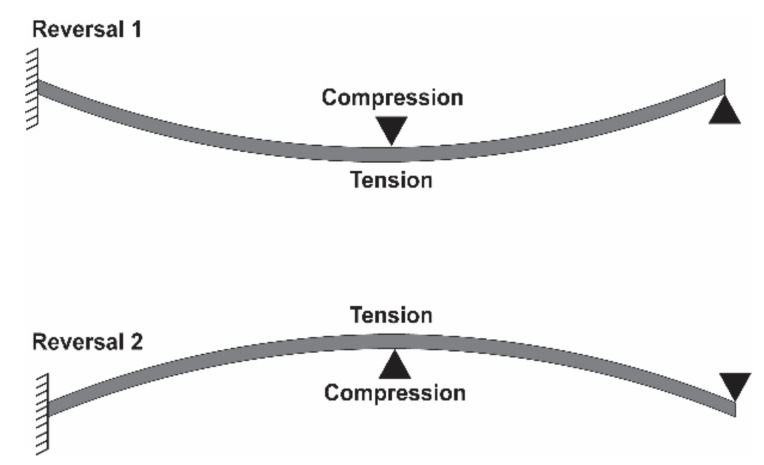
Example of a bending load. When a bending load is applied, the object experiences both tensile and compressive forces. During fully reversed cycling, the tensile and compressive forces alternate sides, resulting in the sinusoidal pattern observed in Figure 3.

Ideally, given continuous cycling, the *N_f_* of a material can be predicted by the strain–life method. In the strain–life method, plastic strain is taken into account by the Coffin–Manson equation (Equation (1)) [108,112,113], which can be used to predict LCF behavior. To predict HCF behavior, and to account for the elastic strain of a material, Basquin’s equation can be used given fully reversed loading of the material (Equation (2)) [108,114]; whereby the minimum and maximum stresses on the material alternate, or reverse, between equal values, but opposite signs (e.g., +1 in tension and −1 in compression) creating a sinusoidal stress pattern, such as what may occur when a stretch sensor is exposed to a bending load (Figure 3 and Figure 4) [108,114]. By combining the Coffin–Manson and Basquin equations, the number of cycles to failure can be predicted (Equation (3)) [108,112,113,114]; however, few studies apply a true bending load to stretch sensors [80].

(1) $$\frac{\Delta \varepsilon_{p}}{2}=\varepsilon_{f}^{\prime }{\left(2N_{f}\right)}^{c}$$

(2) $$\frac{\Delta \sigma }{2}=\sigma_{a}=\sigma_{f}^{\prime }{\left(2N_{f}\right)}^{b}$$

(3) $$\frac{\Delta \varepsilon }{2}=\varepsilon_{a}=\frac{\Delta \varepsilon_{e}}{2}+\frac{\Delta \varepsilon_{p}}{2}=\frac{\sigma_{f}^{\prime }}{E}{\left(2N_{f}\right)}^{b}+\varepsilon_{f}^{\prime }{\left(2N_{f}\right)}^{c}$$

where:

$\frac{\Delta \sigma }{2}$ = the stress amplitude;

$\frac{\Delta \varepsilon }{2}$ = the total strain amplitude;

$\frac{\Delta \varepsilon_{e}}{2}$ = the elastic strain amplitude;

$\frac{\Delta \varepsilon_{p}}{2}$ = the plastic strain amplitude;

$\varepsilon_{f}^{\prime }$ = ductility coefficient of fatigue;

*c* = ductility exponent of fatigue (slope of the plastic line);

$\sigma_{f}^{\prime }$ = strength coefficient of fatigue;

*b* = strength exponent of fatigue (slope of the elastic line);

*E* = modulus of elasticity;

$N_{f}$ = number of cycles to failure.

Further, the sensors would not be worn continuously throughout either the basketball or soccer seasons, which theoretically allows the signal and the strain to recover when not in use, but sensors utilizing viscoelastic polymeric materials may experience a residual extension of the polymer following stretch and relaxation (permanent set) or permanent deformation after only a few cycles that may lead to crack propagation [82,89,115,116,117,118]. Additionally, sensors utilizing thermoplastic polymers may begin to incubate cracks in early cycles that propagate into microstructurally small cracks (MSC) in later cycles, which could further propagate into the long crack regime [119,120], resulting in a drift of the signal or catastrophic failure of the sensor. In coated piezoelectric textiles, interfacial debonding of the conductive materials and fabric could occur at some point, while textiles knitted with conductive filaments may experience residual loop stretch [95,104] or fiber pullout during cycling. Additionally, the material and electrical fatigue properties of the sensors can be affected by their loading histories and exhibit both time and strain dependency [11,12,20,30,75,76,78,79,80,82,83,84,85,87,89,90,91,92,94,95,96,97,100,103]. These material properties are microstructurally driven, and without knowledge of the HCF properties of the sensors, the accuracy of applying the strain–life method and the true fatigue properties of the sensors remain unclear.

Both the Coffin–Manson equation (Equation (1)) and the Basquin equation (Equation (2)) were developed for metals [108,112,113,114]. For metals such as steel, as the number of fatigue cycles increases, a transition from the elastic regime into the plastic regime occurs, where failure results. As the microstructural characteristics of metals and metallics have been extensively studied, crack tip propagation, deflection, or arrest can be mathematically predicted. For example, given constant amplitude, fully reversed loading conditions, the elastic/plastic transition cycle for a metal or metallic material can be identified, and the combined Coffin–Manson–Basquin equation can be used to determine the fatigue life of metals and metallic materials [108,121,122,123,124,125,126]. Determining the transition from the elastic to plastic regimes for polymers is difficult, however, because the behavior of a polymer can vary due to its inherent viscoelasticity, chain length discrepancies, temperatures, and loading conditions that may initiate or inhibit relaxation or creep [127,128]; therefore, the use of the combined Coffin–Manson–Basquin equation (Equation (3)) may not accurately reflect such phenomena to predict the fatigue life of a polymer. 

Rather than focusing on the elastic-plastic transition, Rabinowitz and Beardmore [121] have identified four regimes of fatigue behavior for polymers: (1) an initial or incubation regime where the response to the first cycle of loading remains unchanged, (2) a transition regime in which the peak stress declines, (3) a steady-state regime where the new stress–strain relationship is maintained over many cycles, and finally (4) the crack propagation to fracture regime in which the cross-sectional area of the polymer decreases per cycle even with the application of a constant peak stress. Essentially, regime I represents a monotonic overload, while regime II involves the disentanglement and breakage of polymer chains resulting in cyclic softening. In regime III, the chains have disentangled and the microstructure has stabilized. At the end of regime III, cracks are initiated and the transition to stage IV results in long crack failure of the polymer.

Traditionally, fracture mechanics have been applied to polymers to determine their fatigue lives, but fracture mechanics-based approaches only model long crack propagation (regime 4 of Rabinowitz and Beardmore [121]) and assume the presence of an inherent flaw in the material [120,129]. Methods that singularly incorporate either the Coffin–Manson equation or Basquin’s equation have been used to determine the fatigue life of polymers [120,129,130,131,132,133], but the Coffin–Manson equation, in particular, may more accurately predict the fatigue life of a polymer versus fracture mechanics [120,129,132,134]. For example, the multistage fatigue (MSF) model developed by McDowell et al. [119] incorporates modified Coffin–Manson equation to predict the fatigue life for metals, but the model has also been validated for use in predicting the fatigue life of polymers including acrylonitrile butadiene styrene (ABS) copolymer and polycarbonate (PC) [120,129]. Additionally, the MSF model quantifies the regimes of Rabinowitz and Beardmore [121] and predicts fatigue life by summing the number of cycles (1) of crack incubation (2) in the microstructurally small crack (MSC) regime, and (3) in the long crack regime versus focusing only on the long crack regime as in fracture mechanics [120,129].

Further, a modified Coffin–Manson equation (Equations (4) and (5)) has also been developed specifically for stretchable interconnects [135]. Additionally, by elongating the stretchable interconnects at five different magnitudes using a constant strain rate, repeating the experiment five times, and averaging the elongation values, the electrical fatigue life of the interconnects was predicted using power-law fitting [135]; however, whether the modified Coffin–Manson equation could be combined with Basquin’s equation to determine the total fatigue life of material was not investigated.

(4) $$N_{f}=C\times {\left(\Delta \varepsilon_{pl}\right)}^{-n}$$

where:

(5) $$\Delta \varepsilon_{pl}=\frac{\sqrt{2}}{3}{\left[\begin{array}{c} {\left(\Delta \varepsilon_{X}^{pl}-\Delta \varepsilon_{y}^{pl}\right)}^{2}+{\left(\Delta \varepsilon_{y}^{pl}-\Delta \varepsilon_{z}^{pl}\right)}^{2}+{\left(\Delta \varepsilon_{z}^{pl}-\Delta \varepsilon_{x}^{pl}\right)}^{2}\\ +\frac{3}{2}\left(\Delta \gamma_{xy}^{pl^{2}}+\Delta \gamma_{yz}^{pl^{2}}+\Delta \gamma_{zx}^{pl^{2}}\right) \end{array}\right]}^{\frac{1}{2}}$$

*C* = fatigue ductility coefficient

*n* = reciprocal of fatigue ductility exponent

**Figure 5:**
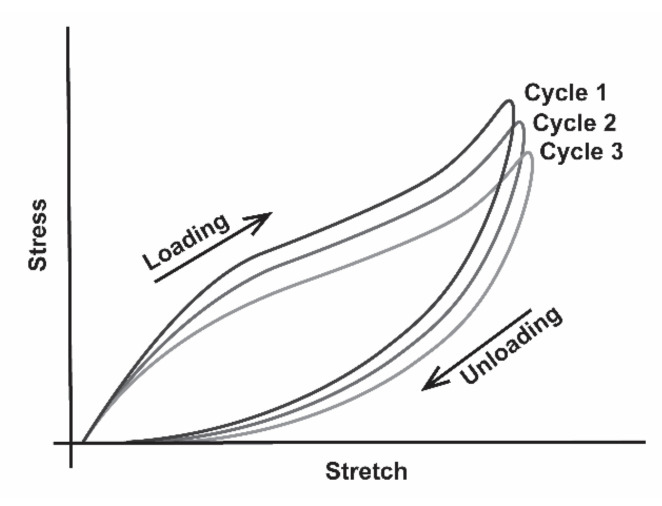
Theoretical example of hysteresis loops indicative of cyclic softening. Cyclic softening occurs when the peak stress of a material decreases with an increased number of cycles.

Equations and models that can be used to predict the fatigue life of a wearable sensor are underutilized, perhaps due to confusion regarding the applicability of an equation or model to a specific sensor. Ultimately, the determination of the appropriate equation or model for predicting the fatigue life of wearable sensors may depend on the loading conditions used during testing [121,132]. Determining the amount of stretch needed to replicate the physiological range of joint motion during mechanical testing is also difficult. As wearable stretch sensors sit atop the skin, Cataldi et al. [136] have argued that mechanical tests should be performed with the sensors stretched to a minimum of 20% strain, which is based on a study by Maiti et al. [137], who measured the skin surface tension of the volar forearm when moved from 90° flexion to 180° extension and found a 26% increase in the principal tensile strain. Although joint movements can be measured, cyclic tests that match the stretch of the sensor to the maximum range of motion (ROM) of a particular joint may underestimate the true fatigue life of the sensors; whereby, LCF testing decreases the time to the onset of plastic deformation [138,139,140]. For example, the polymers utilized in the fabrication of some stretch sensors may be subject to the Mullins effect [116,117,118,141,142,143,144,145]. The Mullins effect occurs when a polymer is loaded in uniaxial tension. Initially, the material exhibits a stiff behavior, but during successive cycles, the hysteresis loops indicate the occurrence of cyclic softening. Cyclic softening is defined by a progressive decrease in stress in response to repeated stretching to the same strain, which decreases the mechanical resistance of the material to deformation (Figure 5). The hysteresis may stabilize provided the strain level in the subsequent cycles does not surpass the strain level reached in the initial cycle, but the softening is permanent. Further, the amount of softening experienced by the polymer is dependent upon both the pre-strain history and the maximum strain to which it is subjected; therefore, at higher strains, such as those produced by stretching to the maximum ROM for a joint, cyclic softening is more pronounced [117,118,142,144,145], and may result in premature failure of the material comprising the sensor.

Due to the variation in materials used to fabricate stretch sensors, the electromechanical properties for the sensors also vary, which has led to different test methods and results. While the materials comprising the sensors may vary, the electrical resistance (*R*) and mechanical strain properties (ε) of the sensors can be correlated by calculating the gauge factor (GF), which measures the sensitivity (*k*) of the sensor (Equation (6)) [4,11,12,13,18,20,30,75,76,77,80,84,85,88,89,90,91,92,93,94,95,96,97,98,99,100].

(6) $$k=\frac{\frac{\Delta R}{R_{0}}}{\frac{\Delta l}{l_{0}}}=\frac{\frac{\Delta R}{R_{0}}}{\varepsilon }$$

where:

ΔR = change in resistance;

$R_{0}$ = initial resistance;

Δl = change in length;

$l_{0}$ = initial length.

An increase in the ratio of the resistance to strain results in a concomitant increase in the sensitivity of the sensor. Further, the gauge factor may be positive or negative. A positive gauge factor indicates an increase in resistance when the sensor is stretched and a decrease in resistance when the sensor is relaxed, while a negative gauge factor indicates a decrease in resistance when the sensor is stretched and an increase in resistance when the sensor is relaxed [92]. As the sensitivities can vary widely depending upon the materials comprising the sensors, the choice of sensor to be used may not only depend on the reproducibility and sensitivity of the signal but may also be application-driven.

Regardless of the materials used in their fabrication, stretch sensors can be defined as either capacitive, resistive, inductive, or having a transistor-like response based on their electrical properties [146], but for wearable applications, capacitive and resistive sensors are the most commonly used [18]. In general, capacitive sensors utilize an insulating material for the sensing mechanism, while conductive or semi-conductive materials provide the sensing mechanism for resistive sensors [146].

Fatigue tests, for both capacitive and resistive sensors, involve multiple cycles of stretching and relaxation to identify whether changes in capacitance or resistance correspond linearly with strain, the magnitude of capacitance or resistance changes, and the sensitivity. Each type of sensor has advantages and disadvantages regarding these electrical properties [18,31,146,147]. Resistive sensors are easily fabricated and integrated into sensor arrays and tend to exhibit high linearity and sensitivity. Disadvantages of resistive sensors include their sensitivity to fluctuations in temperature, non-linearity in the response of the resistance to applied strain, an increase in drift over numerous cycles, and large hysteresis, which compromises the output [18,31,99,146]. Capacitive sensors are relatively stable across a range of temperatures, exhibit relatively low amounts of hysteresis, and do not consume high amounts of power; however, they may be sensitive to electromagnetic interference [18,31,146,147].

Head-to-head comparisons of the fatigue resistance of the stretch sensors are also difficult due to the variety of materials used in in both the fabrication of the sensors and the testing methods employed to assess their fatigue properties. Commonly used materials used to fabricate stretch sensors include polymers, piezoelectric textiles, and carbon nanotubes that are utilized in conjunction with polymers and piezoelectrical textiles, resulting in varying stretch capacities, linearities, and sensitivities depending on the material combinations (Supplementary Table S1).

### 3.1. Fatigue Properties of Polymeric Stretch Sensors

**Figure 6:**
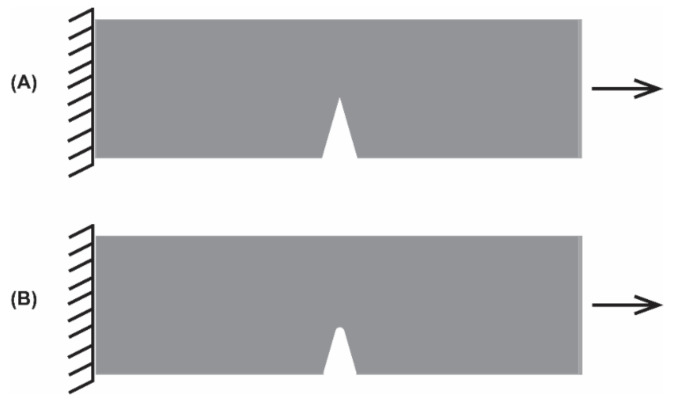
Theoretical examples of crack tips. (**A**) A sharp crack tip promotes rapid propagation of the crack. (**B**) A blunt crack tip slows the propagation of the crack.

As previously mentioned, Rabinowitz and Beardmore [121] defined four regimes to describe the fatigue behavior of polymers. In the initial or incubation regime, the polymer is loaded, while in the transition regime, the peak stress declines (i.e., cyclic softening). During the third steady-state regime, the polymer has adapted to the new stress–strain state, and this regime may be maintained for numerous cycles until cracks begin to develop and the fourth regime, crack propagation to failure, ensues [121]. Depending on the loading history and the maximum strain applied, viscoelastic materials may undergo permanent set, cyclic softening, or microcracks can incubate and begin to propagate [117,118,119,120]. In many materials, including metals, the elastic strain energy driving the microcrack can be dissipated via crack-tip blunting, which can result in arrest of the crack (Figure 6).

Further, the area behind the crack-tip behaves elastically due to the offloading of the strain, and ahead of the crack-tip behaves plastically. While crack-tip-blunting is common in viscoelastic polymers, the elastic strain energy is dissipated by the actual propagation of the crack, which results in plastic deformation behind the crack-tip [115,148]. Additional research has shown that cracks in some viscoelastic polymers, such as silicone, can propagate sideways. In tension, cracks typically propagate orthogonal to the loading direction, but when a crack-tip becomes blunt in some viscoelastic polymers, additional cracks propagate orthogonal to the initial crack. The sideways cracks help dissipate energy and eventually arrest, allowing the crack-tip to sustain the applied load; therefore, the area ahead of the crack-tip remains elastic, allowing for further stretching of the polymer. Sideways cracks were noted to occur in thick samples that underwent tensile testing at low strain rates [148]. Currently, whether other viscoelastic polymers undergo sideways cracking or if the sideways cracks help to extend the fatigue life of such polymers is unknown. Due to their sustained stretchability, viscoelastic polymers are often used in the fabrication of stretch sensors [18]. For example, polymers serve as substrates for thin-film sensors, and polymer tubing and plates are used to house the ionic liquids used in microfluidic sensors. Additionally, the electromechanical properties of hydrogels are being investigated to determine the feasibility of their use as wearable sensors [21,91,97].

#### 3.1.1. Polymer Substrates

Thin films comprised of conductive materials have been tested for their potential use as stretch sensors. To improve their stretchability and durability, polymers are often used as substrates for thin films [11,18,80,85,100]. Under tensile loading conditions, microcracks in the film stretch, and when relaxed, the cracks contract, resulting in a change in the electrical resistance which serves as a proxy for strain [11,80,85]. Initially, the change in resistance follows the overlap model. In the overlap model (i.e., gap-bridging), the edges of the microcracks formed in the thin film are proximal enough to one another to maintain the current [80]. As the microcracks enlarge with subsequent cycling, the resistance increases due to the tunneling effect [100]. The tunneling effect occurs when the current continues to flow between steps that form at the edges of the microcracks and continues until the microcrack propagates into a long crack that results in complete separation of the film [85,119,149]. To cyclically stretch the film without inducing plasticity within the film, either a polymer-based substrate or matrix is utilized because, in addition to their viscoelastic properties, polymer substrates and matrices prevent the occurrence of local strain concentrations within the thin film that could cause the film to fail [11,18,85,100,109].

For example, using bioinspiration, Kang et al. [80] fabricated a sensor comprised of a platinum (Pt) thin film atop a polyurethane acrylate (PUA) substrate. Based upon the strain-sensing crack-shaped slit organ of the spider, the Pt film was bent in a controlled manner to produce cracks with a zig–zag morphology that facilitated gap-bridging; however, even with no strain applied, an approximately 5 nm gap remained between the edges of the crack. Using a strain rate of 0.1 mm/min, the sensors were subjected to 5000 tensile cycles tested at one of two maximum strains, either 0.5% or 2% at a rate of 0.1 mm/min. Sensors tested to a maximum strain of 0.5% exhibited relatively stable resistance for 1000 cycles, after which the resistance began a downward drift; whereas, sensors tested to a maximum strain of 2% exhibited relatively stable resistance for 500 cycles before the resistance began to drift downward. While the resistance exhibited strain and time dependence, the sensor exhibited high sensitivity with a gauge factor of 2079 at strains up to 2% [80].

Due to its stretchability, elasticity, and modulus match with human skin, polydimethylsiloxane (PDMS) is one of the most commonly used polymers in the fabrication of stretch sensors [11,75,81,85,89,100,140,150,151,152,153,154,155,156,157,158,159,160]. Lee et al. [11], Yang et al. [85], and Zou et al. [100] have each fabricated a stretch sensor comprised of a thin film that utilizes a PDMS substrate. Lee et al. [11] performed fatigue tests on a strain sensor comprised of a thin film of silver nanoparticles on PDMS, while both Yang et al. [85] and Zou et al. [100] utilized gold thin films in their stretch sensors. Each of the studies involved 1,000 cycles of stretch and release, but sample dimensions and maximum stretches differed [11,85,100].

The sensor fabricated by Lee et al. [11] utilized silver nanoparticles as the conductive material and was tested at a strain rate of ±2.5%/s for 8000 s with a 10% maximum strain. The maximum change in normalized resistance during fatiguing was measured as 0.24 Ω. Further, to determine the amount of drift incurred by the sensors, a strain rate of 10% was applied to the sensors for 10 min in one test and then for 60 min in another. Both tests were performed at a 10% constant strain. In the 10 min test, drift was measured at 1%, while drift was measured at 1.5% in the 60 min test. A maximum gauge factor of 2.05 at 20% strain was calculated for the sensor [11].

The gold thin-film sensor of Yang et al. [85] was strained to a maximum of 5%, and a maximum gauge factor of 5000 was obtained between 0.7% and 1.0% strain. Resistance varied throughout the cycling, indicating that the resistance was time and strain-dependent. Further, the sensor experienced 20% drift during cycling [85]. Conversely, the gold film sensor fabricated by Zou et al. [100] also included a wavy graphene oxide layer that was overlain by the gold thin film. The sensor was exposed to a varying 19–25% strain during the 1000 cycles. Changes in resistance were induced by the formation and closure of microcracks on the graphene oxide layer. A maximum gauge factor of 2585 at 60% strain was calculated for the sensor. Similar to the gold thin-film sensor of Yang et al. [85], the sensor of Zou et al. [100] also experienced a 20% drift of the resistance.

A thin-film-based sensor has also been fabricated by embedding silver nanowires (AgNW) into a colorless polyimide (cPI) to create a thin-film sensor supported by a polymer substrate [83]. This sensor was subjected to 100,000 tensile cycles at a rate of 0.3 Hz, reaching a maximum strain of 2%, but instead of stretching, the sensor was bent, and the response of the outer bending edge was evaluated. The resistance consistently rose throughout the cycling, and after 100,000 cycles, the resistance had drifted 40%, which possibly resulted from the pullout of the AgNW from the cPI [83].

Similar to Lee et al. [11], silver nanoparticles also served as the conductive material in a sensor fabricated by Borghetti et al. [82]; however, the silver nanoparticles were printed via inkjet onto a polyimide (PI) substrate. Initially, the silver nanoparticle/PI sensor was subjected to 60 tensile cycles of 1% strain at a rate of 5.7%/min. Subsequently, the cycling was repeated on the same sensor at 1.5%, 2%, 2.5%, and 3% strain. The subsequent strains correspond to rates of 11.4%/min, 17.1%/min, 22%/min, and 34.3%/min, respectively. Each 60-cycle set was separated by a 10 min interval. As the number of cycles increased, the resistance decreased, and this trend was pronounced at higher strain amplitudes. The opposite trend was noted for the gauge factor, where the gauge factor increased concomitantly with both the number of cycles and the strain amplitude; therefore, at low strain amplitudes, the silver nanoparticle/PI sensor exhibited reduced sensitivity. The authors also note that the sensor was unable to fully recover from the applied strain [82], indicating the presence of residual strain in the PI.

In addition to PDMS, silicone, and PI, thermoplastic polyurethane (TPU) has also been utilized as a substrate and matrix for conductive materials. For example, Jun et al. [103] deposited AgNW onto one face of a TPU film to create a strain sensor. Using a maximum strain of 30%, the sensor was subjected to 1000 stretch/relaxation cycles at a strain rate of 0.25 mm s^−1^. Similar to Zheng et al. [89], the viscoelasticity of the TPU caused instability in the resistance. Between cycles 0 to 100, the resistance rose sharply with stretching, and after 100 cycles, the resistance somewhat leveled, but continued to slowly rise until exhibiting linearity between cycles 600 and 900. After 900 cycles, the resistance again began to drift upward. In addition to the viscoelasticity of the TPU, the authors note that buckling of the AgNW occurred, which further introduced instability in the resistance and suggests that by depositing the AgNW on both faces of the film, the buckling would be prevented [103].

Losaria and Yim [99] have created a strain sensor that utilizes TPU as a matrix to embed a strain sensor coated in poly(3,4-ethylenedioxythiophene) (PEDOT) and doped with 7 wt. % iron (III) p-toluenesulfonate (FTS). The PEDOT/TPU sensors were subjected to 1000 tensile cycles at a strain rate of 10 mm/min reaching a maximum strain of 10%. Over the duration of the 1000 cycles, the change in resistance was held constant, and the sensors exhibited little hysteresis. Additionally, a gauge factor of >10 at 100% strain was calculated for the sensor [99].

#### 3.1.2. Polymer Housing

Sensors based on the use of ionic liquids as a sensor take advantage of the Poisson’s ratio of polymer-based tubes. As the polymer tube is stretched, its cross-sectional area decreases, deforming the liquid. As the cross-sectional area decreases and the liquids are deformed, a change in resistance occurs [17]. Both Matsuzaki and Tabayashi [71] and Keulemans et al. [88] have utilized these mechanical properties to fabricate ionic-liquid-based sensors housed in silicone tubing.

An ionic liquid comprised of Gallium, Indium, and Tin (GaInSn) was utilized in the sensor fabricated by Matsuzaki and Tabayashi [81]. The sensor was cycled from 0% to 30% strain 20 times at a rate of 10 mm min^−1^ and exhibited little hysteresis, resulting in a linear resistance. Keulemans et al. [88] utilized the ionic liquid 1-butyl-1-methylpyrrolidinium bis (trifluoromethylsulfonyl) imide ([BMPyr][NTf_2_]) in the fabrication of a sensor. This sensor was subjected to several tensile tests, including stretching in 1 mm increments until 10 mm of displacement was achieved. At 10 mm displacement, the sensor failed, having stretched to 400% its original channel length of 2.5 mm. The sensors exhibited a linear response at an average gauge factor of 1.99 at strains ≤ 40% and were sensitive within a 10–25 kHz range. Hysteresis was examined by subjecting the sensor to 40 cycles of stretching to 200%. Initially, the sensor exhibited a large hysteresis of 59% of full scale, but the hysteresis somewhat stabilized after 20 cycles, dropping to 7% of full scale [88]; however, based on the hysteresis curves, the sensor was beginning to undergo cyclic softening. 

In addition to tubing, plates comprised of silicone have been utilized to encase ionic liquids. For example, Choi et al. [13] created a sensor comprised of a rectangular silicone channel with wavy internal sidewalls that housed a mixture of ethylene glycol (EG) and sodium chloride (NaCl). Using a strain rate of 10% s^−1^, sensors were tested at a maximum strain of either 100%, 200%, or 300%. The sensor tested at 100% withstood 10,000 cycles, while the sensor tested at 200% maximum strain withstood 5000 cycles. Finally, the sensor tested at a strain of 300% withstood 3000 cycles. Linearity of the resistance was maintained across all cycles at all maximum strains tested. A gauge factor of 4 was recorded at 250% strain [13], suggesting that the sensitivity of the sensor increased at increased strains.

#### 3.1.3. Hydrogels and Ionogels

Hydrogels have also been proposed for use as stretch sensors. While hydrogels have the advantages of good conductivity, high stretchability, and quick recovery, the mechanical properties and fatigue resistance of some hydrogels are not ideal for long-term sensing applications. The mechanical properties of hydrogels depend on both their chemical constituents and method of polymerization [21]. To improve their mechanical properties, a dual-physically cross-linked double network has been proposed. Specifically, a hydrophobically associated polyacrylamide-hollow latex particles/alginate–calcium (HPAAm-HLPs/Alginate-Ca^2+^) hydrogel was subjected to 10 tensile cycles at a crosshead speed of 80 mm/min. The hydrogel was stretched 200%, 400%, 600%, 800%, and 1000%, exhibiting a rapid recovery time, and the authors argue that the hydrogel also exhibited good fatigue resistance; however, a maximum stress of ~320 kPa was obtained in the first cycle, but by the tenth cycle, a maximum stress of only ~250 kPa was obtained [21], indicating that the hydrogel was susceptible to cyclic softening. 

The strain dependence of hydrogels was illustrated in fatigue tests performed on polypyrrole/PAAm hydrogels fabricated by Chen et al. [91]. Each hydrogel was exposed to one of three maximum strains: 200%, 400%, or 800%. At the strain of 200%, the hydrogel was still intact after 15,000 cycles; however, as the maximum strain was increased, *Nf* decreased. The sensor stretched 400% failed after 5000 cycles, and the sensor stretched to 800% failed after only four cycles. Additionally, the resistance remained linear between 0% and 200% strain, but at higher strains, the resistance began to drift [91].

While typically used for applications such as energy storage [74], ionogels are being investigated for their potential use as stretch sensors [97]. The fatigue properties of a poly (acrylic acid) (PAA) hydrogel immersed in the ionic liquid, 1-ethyl-3-methylimidazolium dicyanamide ([EMIm] [DCA]), were investigated by stretching the resultant ionogel to a maximum of 100% strain over 1400 cycles at a strain rate of 20 mm/min. While the sensitivity increased with increasing strain, the resistance fluctuated during cycling [97].

### 3.2. Fatigue Properties of Piezoelectric Textiles

Numerous studies have focused on the potential use of piezoelectric fabrics or textiles as wearable strain sensors. A piezoelectric textile can be produced by weaving, embroidering, or knitting conductive fibers into the textile [76] or by coating the textile in a conductive coating. Similar to sensors utilizing polymer-based tubes, the Poisson’s ratio of textiles is utilized to create changes in the resistance when stretched. Additionally, as with polymer-based sensors, the mechanical properties of wearable strain sensors can vary based on the fabric substrate chosen for the sensor [78]. The Poisson’s ratio and electromechanical properties of textiles are dependent upon their tightness factor, which is based upon the interlocking loop length used in the textile (Equation (7)) [76].

(7) $$\frac{\sqrt{Tex}}{l}$$

where:

*Tex* = linear density of fibers;

*l* = loop length.

Further, the tightness of a fabric also affects its contact resistance which can be determined through a modified version of Holm’s contact theory equation (Equation (8)) [15,76,95,161]. Typically, higher tightness factors occur in fabrics with a high density of interlocked loop columns (wales) and rows (courses) which decreases the spacing between wales and courses while concomitantly increasing contact pressure and contact points between the two. Higher contact pressure results in lower resistance, but when the textile is stretched, the contact pressure and contact points decrease, increasing the resistance allowing the textile to act as a strain sensor [76].

(8) $$R_{c}=\frac{\rho_{c}}{2}\sqrt{\frac{\pi H}{nP}}$$

where:

$R_{c}$ = contact resistance;

$\rho_{c}$ = contact electrical resistivity;

π = mathematical constant;

H = material hardness;

n = number of contact points;

P = contact pressure.

#### 3.2.1. Conductive Fibers

To examine the effect of the tightness factor on the electromechanical properties of strain-sensing fabrics, Atalay et al. [76] knitted silver-plated nylon yarn into a textile using three different configurations. The first fabric utilized 800 decitex elastomeric yarns knitted at a tension of 0.125 cN/Tex, resulting in a tightness factor of 1.84. While the second fabric also utilized 800 decitex elastomeric yarns, the yarns were knitted at a tension of 0.062 cN/Tex, which resulted in a tightness factor of 1.43. The third fabric utilized 570 decitex elastomeric yarns knitted to a tension of 0.125 cN/Tex and had a tightness factor of 2.17. Each fabric was strained 40% and held for 2 minutes, then released to 0% strain and held for 2 additional minutes before being subjected to 1000 tensile cycles at a maximum strain of 40% using a strain rate of 120 mm/min. The resistance remained stable throughout the 1000 cycles with minimal drift; however, when the strain level remains constant, the higher tightness factor of fabrics 1 and 3 was found to increase error in the resistance when the fabric was relaxed. Each fabric also had varying gauge factors. The gauge factor for fabric 1 was calculated as ~3.75 for strains less than 19%, and 2.16 at strains between 19% and 40%, while fabric 2 had a gauge factor of 4.3 below 9% strain, and 0.9 at strains between 9% and 40%. Finally, fabric 3 had a gauge factor of 0.75 across the entire applied strain range of 0% to 40% [76]. Based on the gauge factors, as the strain increased in fabrics 1 and 2, the sensitivity of the sensor decreased, indicating the capacitance exhibited strain dependence.

A common process of knitting piezoelectric fabrics involves the looped-conductor method. In this method, conductive threads are knitted to form rows of connected loops within the textile [78]. When a tensile force is applied parallel to the rows, the loops are stretched, increasing the resistance. Conversely, when the force is released, the loops return to their original state, and the resistance decreases [14,78]. The looped-conductor method has been used by Gioberto and Dunne [78] to integrate silver-coated nylon threads into either 100% polyester jersey knit, 60% cotton/40% polyester jersey knit, 94% cotton/6% spandex jersey knit, 90% polyester/10% spandex jersey knit, or 82% nylon/18% spandex jersey knit. All samples were stretched 40% and relaxed over 18 cycles, and each sample exhibited linear behavior. Of the five samples tested, the 90% polyester/10% spandex jersey knit exhibited the highest electrical resistance, and the 60% cotton/40% polyester jersey knit the least. In terms of maximum stretchability, the 82% nylon/18% spandex jersey knit exhibited the highest amount of stretch, while the 90% polyester/10% spandex jersey knit exhibited the least amount of stretch. The 90% polyester/10% spandex jersey knit also exhibited the highest hysteresis, and the 100% polyester jersey knit along with the cotton/40% polyester jersey knit both exhibited the lowest hysteresis [78].

Individual multifilaments constructed of polyurethane/poly (PEDOT: poly(styrenesulfonate)) (PU/PEDOT:PSS) knitted into a 15 cm × 10 cm textile swatches have also been subjected to cyclic tensile testing to determine their feasibility as strain sensors [92]. First, individual multifilaments were stretched until failure, resulting in a modulus of 142.8 MPa, a tensile strength of 76.3 MPa, a breaking strain of 414.8%, and a toughness of 145.3 MJ m^−3^. Based on the results of the monotonic tests, the knitted textiles were stretched and relaxed at a strain rate of 20 mm min^−1^ for 500 cycles. The resistance remained stable throughout the 500 cycles with no material failure [92].

Fibers constructed of AISI 316L (low-carbon) stainless steel and polyester were knitted into one of four jersey fabrics with varying wales, courses, stitch densities, and loop lengths to create stretch sensors [95,104]. Each sensor was subjected to six total tensile tests. First, following a resting period of 20 h, the sensor was preconditioned for 250 cycles at a strain rate of 9.6 mm/s and a current of 1 mA. Next, the sensor was allowed to rest for 5 minutes before being subjected to an additional 250 cycles at the same strain rate and current. These testing conditions were then repeated twice, changing only the current used, for a total of three tests. In the first repeated test, the current was raised to 3 mA, and in the second repeated test, the current was raised to 6 mA. The entire testing process was then repeated at a strain rate of 12 mm/s resulting in an additional three tests for a total of six tests per sensor. In general, as the sensors were relaxed to 0% strain, the loops in the fabric remained stretched and required a longer period for the resistance to recover during the preconditioning phase. During the post-conditioning cycling, fewer cycles were necessary to stabilize the resistance. Additionally, recovery time for the resistance was reduced at the higher strain rate of 12 mm/s irrespective of the current applied. Finally, as the number of cycles increased, the fabrics began to loosen, resulting in a decrease in the gauge factor [95,104]. The results of these tests are, however, not unexpected as sensors may require a “break-in” time for the resistance to stabilize [80,103]. Further, the 316L SS/polyester/jersey fabric sensor utilized in the study seemed to be subject to the effects of residual strain as the fabric was described as loosening as the number of cycles increased while concomitantly decreasing the sensitivity of the sensor.

Similarly, knit stretch sensors composed of either a 72% nylon/28% spandex/proprietary conductive polymer, a silver-plated yarn, or a spun stainless-steel yarn were sewn into various fabrics and fatigue tested to determine their feasibility in measuring the movements of the elbows of dancers [98]. To determine the extension value for testing the fabrics, the maximum length of the bent elbow was measured on three people, resulting in a mean value of 60 mm. Each test began with the sample under 10% strain. Samples were then stretched from 110 mm to 170 mm and relaxed at a speed of 6 mm/s for 100 cycles [98]. Using the results of the tests, Liang et al. [98] fit a line to the tensile and the relaxation portions, respectively, of the aggregated cyclic curves for each fabric and calculated the root mean square error (RMSE) of each line. The difference between the error of the lines was then compared to the average error of the sensor to determine the initiation of fatigue into the sample. The higher the error, the more inaccurate the sensor is considered to be in its measurements of movement. Error for most of the samples stabilized after 10–20 cycles; however, error tended to increase for most of the sensors after 75 cycles. Four samples, which included a 100% silver-plated yarn sensor, a silver-plated yarn/100% nylon sensor, a spun stainless steel yarn sensor, and a spun stainless-steel yarn/100% nylon sensor, exhibited fluctuating error values that remained unstable until the samples began to fatigue at 80 cycles. Two of the silver-plated sensors, one comprised of 35% silver fiber/40% cotton/25% polyester knitted in a double bed interlock, and the other comprised of 100% silver fiber in a single bed jersey knit, exhibited no signs of fatigue after 100 cycles [98].

In a fusion of polymers and fabrics, silicone has also been interdigitated with a silver-plated nylon/elastomer blend to create a strain sensor with a gauge factor of 0.83. The sensor underwent 500 tensile cycles at a maximum strain of 50%, and the capacitance experienced 2.9% drift [90].

#### 3.2.2. Conductively Coated Textiles

Strain sensors utilizing a conductive fabric created through a coating of polypyrrole were subjected to 1200 tensile cycles to examine their potential use to gather movement data when placed in a glove worn by patients with rheumatoid arthritis [12]. Samples underwent sinusoidal movements from an amplitude of 1 mm to 5 mm at a frequency of 0.25 Hz. Following the initial calibration, a linear relationship between resistance and stretch was found, but resistance decreased from 43 kΩ during the first cycle to 30 kΩ in the last cycle [12].

A textile comprised of 95% modal/5% spandex and coated with poly(3,4-ethylenedioxythiophene) (PEDOT) underwent 500 cycles of stretch/release at 20% strain to determine its suitability as a strain sensor [96]. Consistency between the strain and resistance profiles over the 500 cycles was noted with a resistance of 0.3 kΩ obtained when stretched to 20% strain and a resistance of 10 kΩ obtained when unloaded (0% strain). An optimal gauge factor of 54 was found at 1.5% strain. Additionally, based on scanning electron microscopy (SEM) images taken after the testing, the PEDOT coating remained intact [96].

Similar to Atalay [90], Yokus et al. [86] have also fused polymers and fabrics to fabricate a stretch sensor; however, instead of utilizing conductive fibers, Yokus et al. [86] utilized a conductive ink printed onto a TPU substrate. Specifically, the construction of the sensor incorporated an 87% polyester/13% spandex fabric overlain by a film of TPU. The Ag/AgCl conductive ink was printed in a meandering pattern on top of the TPU film, and the printed pattern was then encapsulated in an additional TPU film. The fabric/TPU/conductive ink sensor was subjected to 1000 tensile cycles at a strain rate of 10.16 cm/min. A pre-strain of 10% was applied to the sensor, and a maximum strain of 10% was utilized during the cycling. The resistance of the sensor experienced upward drift and plateaued near the end of the 1000 cycles as the resistance of the Ag/AgCl ink began to decrease [86].

#### 3.2.3. Fabric Substrates

**Figure 7:**
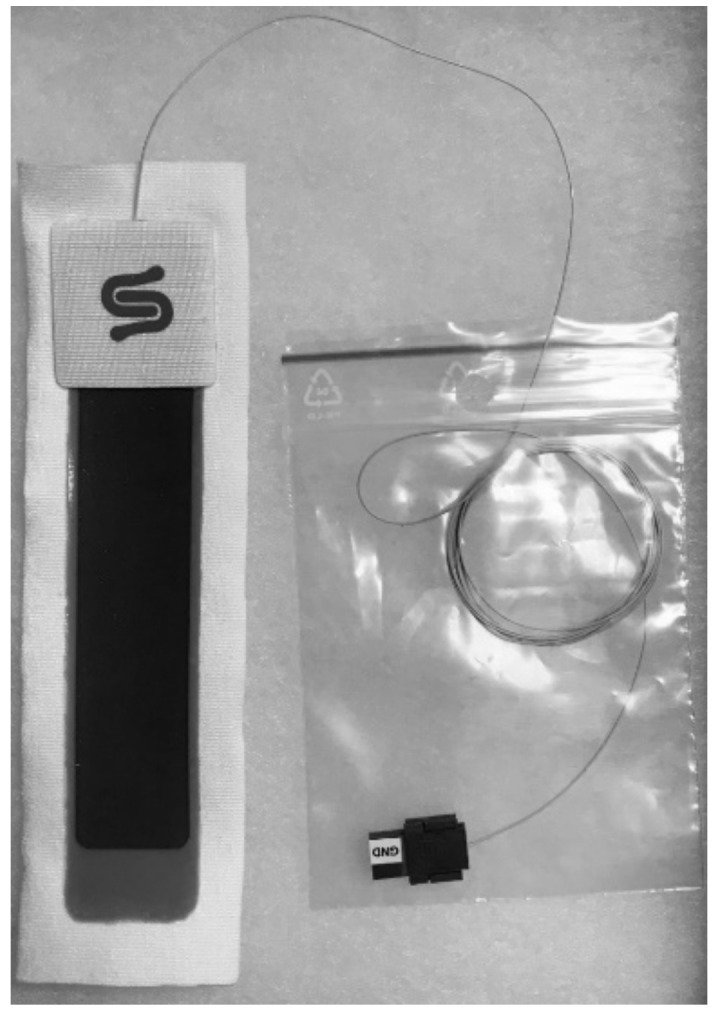
StretchSense™ StretchFABRIC sensor hasa fabric substrate. The polymer housing that protects the sensor is affixed to the substrate via an adhesive.

Stretch sensors such as the StretchSense™ StretchFABRIC sensor are affixed to a fabric substrate to allow for integration into textiles (Figure 7). The majority of fabrics utilized for wearable stretch sensor substrates are based on synthetic polymers due to their strength, stretchability, and moisture-wicking properties [12,30,76,78,86,90,92,95,162,163,164]. Additionally, polymer-based yarns, such as spandex and LYCRA^®^, have been used as substrates for polymer-based stretch sensors to serve a deliberate purpose—strain limitation [165,166]—to prevent a monotonic overload of the sensor. While stretchability is necessary for strain sensors to be able to signal, repeatedly applying or removing the strain sensor could result in an extensive stretch that could damage the sensor; therefore, by adding a fabric substrate, the amount of stretch that can be achieved by the sensor is limited by its fabric substrate, reducing potential damage to the sensor during application or removal [165,166].

Cotton is an especially attractive option for wearable medical and telehealth sensors because cotton is hypoallergenic [167], ecofriendly [168,169,170], lightweight [170], cost-efficient [168,170,171], sustainable [168], and easily laundered [169,171]. In addition to these properties, cotton serves as a “blank canvas” that may be treated with antimicrobial agents—a property that is desirable when monitoring wound healing [167,172,173,174,175,176,177,178,179].

While cotton has potential for use in wearable sensors, cotton as a substrate has some drawbacks. Specifically, cotton fibers lack natural wicking properties, stretchability, and strength in comparison to synthetic fibers [78,162,170,180]. Additionally, at stretches in excess of 10%, cotton may incur permanent deformation of its loop structure [180]. Conversely, synthetic fabrics, such as spandex, still maintain their loop structures for 30% stretch [180]. Finally, cotton does not exhibit the tensile strength or elasticity of synthetic fibers such as nylon [164,170]. Such drawbacks, however, are generally based on the use of cotton in sportswear or in stretch sensing applications, but cotton fabrics have been used as substrates for health care monitoring. For example, because of its low price point, flexibility, launderability, and light weight, cotton has been used as a substrate for different wearable electrocardiograms [181,182,183,184,185,186].

If a stretch sensor is to be affixed to a fabric substrate, the intended use of the sensor and fatigue properties of the substrate should also be considered. While fabric substrates may be purposefully used to limit the tensile strains placed on a stretch sensor [165,166], such limitation may not always be desirable, and repeated cycling of the stretch sensor may result in a failure of the fabric substrate [187].

### 3.3. Fatigue Properties of Carbon Nanotube Stretch Sensors and Graphene Stretch Sensors

Due to their inherent conductivities and stretchabilities, CNTs and graphene are attractive options for use in stretch sensors. As with polymers and fabrics, both CNT- and graphene-based sensors also use Poisson’s ratio to induce changes in the conductivity of the sensors. Initially, applied strain causes the conductive network created by CNTs to deform or break, creating islands and gaps, which may be spanned by some CNTs, in the network, increasing the resistance. As the strain is removed, the conductive network reorganizes and resistance decreases. Eventually, the reorganized network stabilizes, allowing the resistance/capacitance to also stabilize; therefore, sensors utilizing CNTs may require a break-in period to stabilize the electromechanical response [75,87,188]. Similarly, graphene-based sensors utilize a connection–disconnection property to effect conductive changes. At 0% strain, the graphene sheets overlap and are in contact with one another, but when a strain is applied, the graphene sheets become disconnected, and the resistance increases [147]. One major drawback of using CNTs and graphene should be noted—both are toxic. CNTs are toxic, carcinogenic, teratogenic, and embryolethal [189], while exposure to graphene can result in the disruption of cellular processes and pathways, including the production an inflammatory response, the damaging of DNA, and the induction of apoptosis [190].

#### 3.3.1. CNTs with Polymer Substrates

PDMS has also served as a substrate for CNT-based sensors. For example, a single-wall carbon nanotube (SWCNT) thin-film strain sensor fabricated by Yamada et al. [75] utilized a PDMS substrate. A maximum gauge factor of 0.06 was recovered for the sensor at strains between 60% and 200%. Three sensors were fatigued at a strain rate of 6 mm s^−1^. Sensor 1 was tested at a maximum strain of 100%, while Sensor 2 was strained to 150%, and finally, Sensor 3 was strained to 200%. Sensors 1 and 2 completed 10,000 cycles, but Sensor 3 completed only 3300 cycles due to a catastrophic failure of the PDMS substrate. A drift of 6% was reported for the resistance [75].

Zheng et al. [89] have also fabricated both a CNT sensor and a carbon black (CB) sensor that utilize a PDMS matrix. Each sensor was subjected to 100 cycles of tension/relaxation at a maximum strain of 10% using a rate of 0.033 Hz. The gauge factor for the CB/PDMS sensor was higher (15.75 to 10% strain) than that of the CNT/PDMS sensor (4.36 to 10% strain). Additionally, the resistance for both sensors exhibited drift, and the authors note that the resistance never returned to its initial value when relaxed to 0% strain due to the destruction of the connective networks within the sensor and the viscoelastic behavior of the PDMS [89].

A sensor comprised of entangled SWCNTs wrapped in a thermoplastic elastomer to create a coaxial fiber has been examined for its use as a strain sensor that can be incorporated into textiles [93]. Similar to film-based sensors, microcrack formation of the carbon nanotubes during loading results in a change in the resistance. Using an Instron 5944, the sensor was subjected to 3250 tensile cycles (tension/relaxation) ranging from 20 to 100% strain at a strain rate of 400 mm/min^−1^. Resistance remained stable over the 3250 cycles, peaking at ~5 × 10^6^ Ω/cycle when stretched to 100% strain. A gauge factor of 48 was calculated for 0–5% strain, and at 20–100% strain, a gauge factor of 425 was calculated. Hysteresis loops were also presented, but only for five tensile cycles ranging from 0 to 100% strain. The hysteresis for the five cycles was stable [93].

Silicone was utilized as a substrate for a thin-film sensor comprised of both single- and double-walled carbon nanotubes (CNT) [77]. Similar to the sensor tests performed by Yamada et al. [75], Cui et al. [77] performed three fatigue tests on their sensors at a strain rate of 10 mm/s, but the sensors were cycled to failure. In one test, the sensor was stretched to a maximum of 100%, and in another test, the sensor was stretched to 150%. The final test involved stretching the sensor 200%. In each of the three tests, a “step and hold” test was performed every 1000 cycles, whereby the sensor was strained to the maximum stretch for the test at a rate of 5 mm/s, held for 10 s, and released. The capacitance for each tested sensor remained fairly stable over the cycling. Further, the sensor stretched 200% failed at cycle number 1800, while the sensor stretched 150% failed at cycle 3800. Finally, the sensor stretched 100% failed at cycle 10,000. The authors, however, note that the sensors failed near the grip section, suggesting that the sensors may have a longer fatigue life than is indicated by the tests [77].

A multiwalled carbon nanotube/Epoxy (MWCNT/EP) strain sensor created by Cao et al. [87] exhibited strain-dependent effects on the resistance. The MWCNT/EP sensor was stretched and relaxed over 10 cycles at a crosshead speed of 0.5 mm/s. At the lower strains of 2% and 4%, the relationship between strain and resistivity was approximately linear, but with increasing strain to 6% and 8%, the resistivity decreased, which indicates that the resistivity is dependent on the strain rate [87].

Using an everyday office item, Wang et al. [1] fabricated a sensor by embedding CNTs into an elastic rubber band (EB) and then coating the band in polydopamine (PDA). Fatigue testing was performed on the sensor using a strain rate of 100 mm min^−1^ and a maximum strain of 100% for 10,000 cycles. The resistance of the sensor slowly drifted downwards until cycle 50 and was attributed to the damaging and restructuring of the CNTs. After 50 cycles, the resistance began to slowly drift upward until it stabilized at approximately cycle 5000. Between cycle 5000 and 9000, the resistance remained fairly stable, after which the resistance began to drift upwards again [1].

#### 3.3.2. CNTs Incorporated in Piezoelectric Textiles

MWCNTs have also been investigated for their strain-sensing abilities. Zhang et al. [79] fabricated an MWCNT/spandex strain sensor. At a strain rate of 100%/min and a maximum strain of 5%, the sensor was subjected to 80 stretch/relaxation cycles and found that the resistance showed time and strain dependency [79].

#### 3.3.3. Additional CNT Sensors

Strain sensors have also been fabricated from carbon nanotube meshes (CNTMs). Guo et al. [105] created CNTMs by depositing the CNTs on a nickel mesh. PDMS served as the substrate for the CNTMs. The sensors were tested for 1000 cycles at a maximum strain of 20% and a strain rate of 2% s^−1^. The resistance of the CNTMs sensor remained linear throughout the 1000 cycles; however, similar to the MWCNT sensors, the CNTMs sensor also exhibited strain dependency [79,87,105]. Additionally, as the strain increased, the resistance also increased [105].

#### 3.3.4. Graphene Sensors

As with CNTs, graphene-based sensors have also been used in conjunction with polymers and fabrics. For example, Liu et al. [106] examined the potential of graphene embedded in TPU to create stretch sensors, while Gao et al. [94] fabricated a sensor comprised of a graphene–polyurethane nanofiber composite. In the study of Liu et al. [106], varying concentrations of graphene, including 0.2 wt. %, 0.4 wt. %, and 0.6 wt. % were introduced into a TPU matrix. The resultant sensors were then subjected to 100 tensile cycles at a strain rate of 0.1 min^−1^ to a maximum strain of 50%. Of the three concentrations, the 0.2 wt. % graphene concentration reached the highest resistance, and the authors further found that the 0.2 wt. % graphene/TPU sensor had the highest sensitivity and a homogenous dispersion of graphene throughout the TPU; however, when strained 30%, the resistance did not return to its initial value, potentially due to the presence of residual strain in the TPU matrix or, owing to the large size of the graphene, the graphene takes longer to recover and restore the conductive paths, creating a lag between the mechanical and electrical properties of the sensor [106].

In addition to the graphene–polyurethane nanofiber composite, Gao et al. [94] also introduced quartz into their sensor to improve its electromechanical properties. The sensor was subjected to 600 cycles of tensile strain to 50% at a rate of 30 mm·min^−1^. Following an initial decrease, the resistance somewhat stabilized. A maximum gauge factor of 5.9 was recovered between 94 and 110% strains [94].

White et al. [30] integrated both polymers and fabrics into expanded intercalated graphite (EIG) elastomer composite-based sensors in one of three ways: (1) by creating a conductive film, (2) by screen-printing the conductive materials onto spandex, or (3) by 3-D printing the conductive materials onto a fabric substrate. Reaching a maximum strain of 50%, the sensors were subjected to 100,000 cycles at a strain rate of 80 mm min^−1^. Based on the results of the cyclic tests, all three sensors exhibit a decrease in sensitivity with an increase in the number of cycles; however, the linearity of the sensors remained intact [30].

Using graphene nanoplatelets deposited on a PDMS coated polyethylene terephthalate (PET) film, Wang et al. [191] created a stretch sensor that was subjected to 1000 bending loads. The sensor exhibited linearity to 30% strain with a GF of 36.2. The sensor exhibited minimal electrical hysteresis and rapid response. Additionally, the sensor recovered from the bending fatigue and did not exhibit signs of material failure [191].

## 4. Discussion

No standards currently exist for the fatigue testing of wearable sensing technologies [40,74]. While standards are being developed for internal wearable sensors [74], the methods for testing the cyclic durability of external sensors vary widely. For example, cycling of stretch sensors ranges from 10 cycles [21,87], which is below the low-cycle fatigue (LCF) regime [108], to 100,000 cycles [30,83], which falls within the high-cycle fatigue (HCF) regime [108]. HCF data for stretch sensors is rare [30,83], with most studies focused on LCF data [11,12,13,20,75,76,77,80,82,84,85,86,89,90,91,92,93,94,95,96,97,98,99,100,103]. 

Variation in materials and testing methods coupled with the inconsistent reporting of testing conditions and resultant data not only make direct comparisons of the fatigue life of wearable sensors difficult but also makes replication and validation of the sensor studies difficult. Despite these difficulties, several trends can be gleaned from the data and provide a basis for additional studies: (1) incremental changes in the design or materials comprising ICD leads can have adverse consequences; (2) thin-film and CNT-based sensors tend to have high sensitivities [85,93], but may require a break-in period to stabilize their electromechanical response [1,75,79,80,87,189]; (3) ionic liquids exhibit stable resistance responses, whereby the change from the base resistance to the peak resistance during cycling remains stable [13,81,88]; (4) at low strain amplitudes, CNT, hydrogel, and ionogel sensors are durable with respect to their material properties [75,77,91,97]; and (5) many of the studies assess only the LCF behavior of the sensors and do not cycle to failure [1,11,12,13,21,76,78,79,80,81,84,85,87,88,89,90,92,93,94,95,96,97,98,103,104]. Additionally of note, residual strain or permanent set can create a lag between the mechanical and electrical responses of sensors or cause drift of the resistance [84,89,95]. Additionally, the phenomenon of cyclic softening is underrecognized [21,88] and may ultimately result in the failure of the sensor. Additionally, some of the fluctuation and drift in the resistance/capacitance of the sensors may result from compliance in the testing machines, especially those relying on the crosshead speeds, increasing the uncertainty in the electrical properties of the sensors. Finally, as more wearable sensors become commercially available, the need for a break-in period for some sensors raises questions of whether to apply pre-strain to the materials comprising the sensors or to the completed sensor to reduce errors in resistance and sensitivity, making such sensors operable “out of the box”.

The electromechanical properties associated with stretch sensors are complex, and additional research is necessary to understand their fatigue behaviors and failure mechanisms [139]. In particular, both HCF and failure data for wearable sensing technologies are needed. The collection of HCF data will not only help to determine the fatigue lives of the sensors but will also help to determine, modify, or develop equations and models that can then be validated for use in predicting the fatigue life of similar sensors. Further, fractographic analyses of failed sensors would further the understanding of when cracks are initiated and how they propagate throughout sensors comprised of various combinations of materials.

By recognizing the electromechanical fatigue behaviors associated with the materials used in the fabrication of the sensors, durable sensors can be crafted. Recognition of phenomena associated with the fatigue properties of particular materials will allow sensor researchers and manufacturers to choose materials that best suit the purpose of their particular sensor. Additionally, the development of standards for the fatigue testing of wearable sensors [40,74,187] is needed and will allow for consistent reporting of testing methodologies and output. The development of standards will ultimately improve the ability of researchers, manufacturers, health professionals, and consumers to perform head-to-head comparisons of wearable sensing technologies.

## Supplementary Materials

The following are available online at https://www.mdpi.com/article/10.3390/ma14154070/s1, Table S1: Sensor fatigue literature review results.
